# Genotoxicity test of eight natural color additives in the Korean market

**DOI:** 10.1186/s41021-022-00247-0

**Published:** 2022-06-08

**Authors:** Byungkyung Do, Hoonjeong Kwon

**Affiliations:** 1 Department of Food and Nutrition, Seoul National University, Gwanak-ro 1 Gwanak-gu, Seoul, 08826 Republic of Korea; 2 Research Institute of Human Ecology, Seoul National University, Seoul, 08826 Republic of Korea

**Keywords:** Natural color additives, Ames test, In vitro chromosomal aberration test, In vivo alkaline comet assay, *Curcuma longa* Linne extract, *Monascus* red

## Abstract

**Background:**

Various natural color additives are preferred by many consumers over synthetic color additives because they are perceived to be safer. However, most do not have sufficient toxicity data for safety assurance. Color ingredients in particular have some structures suspected of being toxic. Eight natural color additives, *gardenia* red, blue, and yellow; lac color; cochineal extract; beet red; *Curcuma longa* Linne extract (*Curcuma* extract); and *Monascus* red, currently permitted for use in Korea, were selected and subjected to genotoxicity tests. Acceptable daily intake values have not been allocated to these color additives (except for cochineal extract) due to the lack of toxicity data. We used genotoxicity testing—the bacterial reverse mutation test (Ames test), in vitro mammalian chromosomal aberration test, and in vivo alkaline comet test—for minimum safety assurance.

**Results:**

*Gardenia* red and blue, cochineal extract, lac color, and beet red did not induce mutagenicity or chromosomal abnormalities. *Gardenia* yellow was mutagenic in the Ames test, but was not positive in the in vitro chromosomal aberration test or in vivo alkaline comet assay. *Curcuma* extract and *Monascus* red induced cytotoxicity in the Ames test at high concentrations in *Salmonella typhimurium* TA1537 and TA100, without showing mutagenicity. On cytotoxicity testing, *Curcuma* extract and *Monascus* red showed cytotoxicity at concentrations higher than 313 μg/ml in Chinese hamster ovary CHO-K1 cells and showed equivocal results in chromosomal aberration assay of the same cells. *Curcuma* extract and *Monascus* red produced significant increases in DNA damage at a dose of 2000 mg/kg b.w./day, and induced dose-dependent increases in % DNA in the tail and tail moment on in vivo comet assay.

**Conclusions:**

Six out of eight food colorants did not cause genotoxicity and cytotoxicity. However, *Monascus* red and *Curcuma* extract showed definite cytotoxicity and probable genotoxicity.

## Introduction

Food additives are essential to the food industry, and food additives of natural origin are preferred by many consumers because they are perceived to be safer than their synthetic counterparts [1–3]. In Korea, if a natural additive is derived from a plant with a history of safe use in food, one that causes no toxic or adverse effects, then the additive may be deemed safe for use or limited use in food without toxicity testing [4]. Botanical food additives derived from sources with a long history of safe use are also considered safe by foreign regulatory agencies, such as the European Food Safety Authority (EFSA) and the U.S. FDA (Food and Drug Administration) [5, 6]. However, several reports have alleged that such waivers of toxicity testing for ingredients with a history of use have caused safety problems. In 1968, calamus, calamus oil, and its extract, which had been used as flavorings, were prohibited by the FDA [7]. Beta-asarone, an active ingredient in calamus oil, was carcinogenic in rodents and caused intestinal cancer ( [8]). Safrole is another food ingredient that was later banned. Sassafras oil, which contains safrole, was used as a flavor in soft drinks such as root beer. However, as a food additive it was banned in the United States in 1960 because safrole causes liver cancer [9, 10]. Madder (*Rubia tinctorum* L.), a natural color additive, had been in use in Korea and Japan for many years, but experiments revealed that Madder additives have potential nephrocarcinogenicity and mutagenicity in humans, and their use as food additives was banned in 2004 by the Korean MFDS (Ministry of Food and Drug Safety) [11–13].

Longstanding use cannot guarantee the absence of genotoxicity. Unlike other types of toxicity, the causal relationship between exposure to a genotoxic substance and phenotype expression is difficult to determine because expression of genetic alterations can only be observed long after exposure to the substance [14–16]. Furthermore, whereas most toxic endpoints have a threshold, genotoxicity is deemed to occur at even low exposure levels. Although recent efforts have sought to explore whether thresholds can be established even for genotoxic substances, which have historically been assumed to pose health risks irrespective of their dose levels, regulatory agencies continue to embrace the no threshold concept [17–21]. Therefore, it is necessary to conduct genotoxicity testing to ensure the safety of natural food additives.

The OECD (Organization for Economic Co-operation and Development) has 13 test guidelines for identifying genetic toxicity. The EFSA recommends a test battery for evaluating the genotoxic potential of food additives, including a list of possible endpoints (i.e., induction of gene mutations and structural and numerical chromosomal alterations) that can be used to deem a substance genotoxic. The studies used to investigate gene mutation are the bacterial reverse mutation test (OECD TG 471, Ames test) and the in vitro mammalian cell gene mutation test (OECD TG 476 or OECD TG 490). The studies used to investigate chromosome aberrations are the in vitro mammalian chromosomal aberration test (OECD 473) and the in vitro mammalian cell micronucleus test (OECD TG 487). The follow-up in vivo tests are the mammalian erythrocyte micronucleus test (OECD TG 474), the mammalian bone marrow chromosome aberration test (OECD 475), the transgenic rodent somatic and germ cell gene mutation assays (OECD TG 488), and the in vivo alkaline comet assay (OECD TG 489) [22]. The Korean MFDS provides options for genotoxicity testing that allow researchers to combine genotoxicity tests in a limited way in the “Standard for Toxicity Study of Pharmaceuticals” [23]. A test battery consists of three tests: the bacterial reverse mutation test (OECD TG 471), one of the in vitro test methods (OECD TG 473, OECD TG 487, or OECD TG 490), and one of the in vivo test methods (OECD TG 474 or OECD TG 475).

In this study, we tested eight natural color additives permitted for use in Korea: *gardenia* red, *gardenia* blue, *gardenia* yellow, lac color, cochineal extract, beet red, *Curcuma longa* Linne extract (*Curcuma* extract), and *Monascus* red. Among those substances, only cochineal extract has an official acceptable daily intake (ADI) value (0–5 mg/kg b.w./day) in Korea, which was set in 2012. However, that ADI was set for carmines, the active ingredient in cochineal extract, by the JECFA (Joint United Nations and World Health Organization Expert Committee on Food Additives). The JECFA did not set an ADI for cochineal extract because toxicity studies were insufficient and specifically stated that an ADI of 5 mg/kg b.w./day could not be applied to cochineal extract. In addition, although genotoxicity testing of cochineal extract did not follow the recommended OECD test methods, the extract did return negative results in a *Salmonella* test and positive results in a chromosomal aberration assay in Chinese hamster ovary cells. Therefore, additional genotoxicity tests on cochineal extract are warranted to further elucidate the genotoxic potential of this substance [24–26]. In 2017, the Korean MFDS chose not to allocate ADIs for *gardenia* red, lac color, and *Monascus* red due to insufficient toxicity data, including genotoxicity data [27]. ADIs for *gardenia* blue and yellow were allocated as “not specified”, but that classification was given without an evaluation of toxicological data [28]. Those test substances had equivocal genotoxic test results. The results for *gardenia* yellow differed in the studies of Ozaki et al. [29] and Chung et al. [30]. *Gardenia* yellow caused DNA damage in a *rec*-assay in Ozaki’s study but did not reveal any positive results in the Ames test or in in vitro or in vivo genotoxicity tests in Chung’s study. *Gardenia* blue showed no genotoxic potential, however, its natural precursor, genipin, demonstrated positive results in the Ames test and in vitro micronucleus assay [31]. Banerjee et al. evaluated the genotoxicity of lac dye in 1984 and found no mutagenicity or cytotoxicity in the Ames test or on in vitro mutation test using Chinese hamster lung cells. However, aberrant chromosomal effects were observed in the in vivo chromosomal aberration assay using mice [32]. The Korean MFDS has not yet set an ADI for beet red or *Curcuma* extract. For beet red, the ADI was allocated as “not specified” by the JECFA and EFSA based on a history of use. Although *Curcuma* extract was evaluated by the JECFA in 2003 and an ADI was allocated, there are results that might not be disregarded on genotoxicity [33, 34]. Curcumin is generally recognized as safe (GRAS) by the U.S.FDA. In GRAS notice no. 822, genotoxicity testing—bacterial reverse mutation test, in vitro chromosomal aberration test, and in vivo micronucleus test—performed by the Laurus Lab using synthetic curcumin (purity > 99%) was cited. While the results of bacterial reverse mutation test and in vivo micronucleus test were negative, in vitro chromosomal aberration test exhibited chromosomal abnormalities. However, some studies have suggested that chromosomal abnormalities were due to hydroxyl radical formation from test conditions, and some previous genotoxicity tests of natural curcumin produced negative results. Taking these results together, the FDA concluded that synthetic curcumin was not genotoxic [35, 36].

For the eight natural color additives we selected as test substances, no clear conclusions have been made about their toxicity, including genotoxicity. In this study, we conducted genotoxicity tests—the bacterial reverse mutation (Ames) test, in vitro mammalian chromosomal aberration test, and in vivo alkaline comet test—to evaluate the safety of these natural color additives. The objective of the present study was to confirm the safety of these “natural color additives”, which are available in markets without an established ADI on the basis of the “history of use”, not chemicals, and to protect those who consume them.

## Materials and methods

### Chemicals and test substances

Color additives were purchased directly from the manufacturers. *Gardenia* blue (color value ${\mathrm{E}}_{1\mathrm{cm}}^{10\%}$ ≥ 270), *gardenia* yellow (${\mathrm{E}}_{1\mathrm{cm}}^{10\%}$ ≥ 600), cochineal extract (≥ 4.5% as carminic acid content), *Monascus* red (${\mathrm{E}}_{1\mathrm{cm}}^{10\%}$ ≥ 1000), beet red (${\mathrm{E}}_{1\mathrm{cm}}^{10\%}$ ≥ 29), and *Curcuma longa* Linne extract (*Curcuma* extract) (${\mathrm{E}}_{1\mathrm{cm}}^{10\%}$ ≥ 780) were purchased from ES Food Co., Ltd. (Gyeonggi-do, Korea). Lac color (${\mathrm{E}}_{1\mathrm{cm}}^{10\%}$ ≥ 89) was obtained from Edentown FNB Co. (Incheon, Korea). *Gardenia* red (${\mathrm{E}}_{1\mathrm{cm}}^{10\%}$ ≥ 60) was obtained from MSC Co., Ltd. (Gyeongsangnam-do, Korea). The quantity for test substances is indicated as a color value (${\mathrm{E}}_{1\mathrm{cm}}^{10\%}$) based on the Korean Food Code, which represents values calculated using the absorbance of 10% solution of the colorant, and the regulatory criteria for hazardous compounds were satisfied. All chemical reagents were purchased from Sigma-Aldrich Korea (Seoul, Korea), Wako Pure Chemical Industries, Ltd. (Osaka, Japan), Moltox (Molecular Toxicology, Boone, NC, USA), or Genogen Co., Ltd. (Chungcheongbuk-do, Korea).

### In vitro mutagenicity assay

#### Bacterial strains

The strains for the bacterial reverse mutagenicity test were chosen using the OECD guideline [37]. *Salmonella typhimurium* TA98 and TA1537 were chosen to detect mutations by frameshift, and *S. typhimurium* TA100 and TA1535 and *Escherichia coli* WP2*uvrA* were used to detect point mutations by base-pair substitutions. Those five strains were purchased from Moltox (Molecular Toxicology, Boone, NC, USA). After we confirmed the genotypes with the strain check assay [38, 39], we stored the culture stocks below − 80 °C. The test strains were prepared by incubating them overnight (10 h) at 37 °C in a nutrient broth (Oxoid No.2) to reach a concentration of 1–2 × 10^9^ bacteria/ml.

#### Dose selection

The maximum test concentration for the bacterial reverse mutation test was 5 mg/plate, as recommended by OECD TG 471 [37]. Five different analyzable concentrations were set at intervals of √10. Each bacterial strain was treated with substances at concentrations of 50, 150, 500, 1500, and 5000 μl/plate. Distilled water was used as a negative control, and the mutagens sodium azide (NaN_3,_ 0.5 μg/plate for TA1535), 9-aminoacridine (9-AA, 40 μg/plate for TA1537), 2-(2-furyl)-3-(5-nitro-2-furyl) acrylamide (AF-2, 0.1 μg/plate for TA98, 0.1 μg/plate for TA100, and 0.01 μg/plate for WP2*uvrA*), and 2-aminoanthracene (2-AA, 0.5 μg/plate for TA98, 1 μg/plate for TA100, 2 μg/plate for TA1535 and TA1537, and 10 μg/plate for WP2*uvrA*) were used as positive controls to verify bacterial susceptibility to established genotoxins.

#### Bacterial reverse mutation assay

Bacterial reverse mutation assays were conducted according to the OECD TG 471 guidelines (OECD, 1997) and using the method published by Maron and Ames [40]. Each strain was treated with substances by concentration, with (+S9) or without (−S9) metabolic activation. Metabolic activation was provided by 0.5 ml of Aroclor 1254-induced rat liver S9 (Moltox, NC, USA), 0.5 ml of distillation, and 9 ml of Cofactor mix (C1 Life Tech, Chungcheongnam-do, Korea). Each 1 ml of S9 mix contained 8 μmol of MgCl_2_, 33 μmol of KCl, 5 μmol of glucose-6-phosphate, 4 μmol of NADPH, 4 μmol of NADH, and 100 μmol of sodium phosphate (pH 7.4). All of the test substances and NaN_3_ were prepared in distilled water, and 9-AA, AF-2, and 2-AA were dissolved in dimethyl sulfoxide. Mutagenicity was assayed using the pre-incubation method. The test substances were added to 0.5 ml of phosphate buffer or S9 mixture and 0.1 ml of bacterial culture and then incubated at 37 °C for 30 min. After this, 2 ml top agar was added to the mixture and poured onto minimal agar. The plates were incubated at 37 °C for 48 h, and the revertant colonies were counted. All experiments were analyzed in triplicate.

### In vitro chromosomal aberration test

#### Dose selection

The maximum test concentration for in vitro chromosomal aberration test was 5 mg/ml, as recommended by OECD TG 473 [41]. In this study, since the test substances were mixtures of unknown composition, the highest concentration was set to 5 mg/ml instead of 2 mg/ml, and then concentrations were set to 2500, 1250, 625, and 313 μg/ml at equal intervals. Before the in vitro chromosomal test, cytotoxicity testing was performed using the five concentrations listed above to determine the concentration that caused cytotoxicity. Distilled water was used as a negative control, and mutagens 4-nitroquinoline-N-oxide (4-NQO, 1.0 μg/ml for short-term treatment and 0.5 μg/ml for continuous treatment without metabolic activation) and cyclophosphamide monohydrate (CPA, 5.0 μg/ml for short-term treatment with metabolic activation) were used as positive controls.

#### Cytotoxicity test

Eight substances were tested for cytotoxic effects using thiazolyl blue tetrazolium bromide (MTT) assay on Chinese hamster ovary CHO-K1 cells. Cells were seeded in 96-well plates at a density of 1 × 10^4^ cells/well. After 24 h of incubation, the cells were treated with test substances, up to 5000 μg/ml, for 24 h at 37 °C in a 5% CO_2_ incubator. The culture medium and test substances were removed, and MTT reagent solution (10 μl) was added to each well, which contained 90 μl of cells in culture medium. Then, the plate was incubated for 4 h at 37 °C. Subsequently, DMSO was added to dissolve the resulting formazan, and absorbance was measured at 540 nm using a microplate reader (SpectraMax iD3, Molecular devices, CA, USA).

#### In vitro mammalian chromosome aberration test

CHO-K1 cells for the chromosomal aberration assay were chosen, CHO-K1 cells were obtained from the Korea Cell Line Bank and cultured in RPMI-1640 medium with 10% fetal bovine serum (Welgene, Korea). Under these conditions, the population doubling time was 15 h. Based on the results of the cell viability assay, the cells were treated for 5 doses up to 5000 μg/ml for *gardenia* red, *gardenia* yellow, beet red, and cochineal extract; 4 doses up to 2500 μg/ml for *gardenia* blue and lac color; and three doses up to 313 μg/ml for *Monascus* red and *Curcuma* extract. The cells were treated with various doses of test substances, a negative control (distilled water), or positive control chemicals (4-NQO and CPA) 3 days after being seeded at 1 × 10^5^ cells/ml in a T25 flask. Exposure durations were 6 h and 24 h without S9 and 6 h with S9. The final concentration of S9 in the cultures was 2%. At the end of the 6 h exposure periods, the treatment media were replaced with complete medium. Colcemid was added to the cultures at a concentration of 0.2 μg/ml 2 h prior to termination of the 24 h culture period. After harvesting, the cells were incubated in a 0.075 mol/L KCl solution for 15 min at 37 °C. Then they were fixed three times with ice-cold methanol/glacial acetic acid (3:1, v/v). The fixed cell suspension was dropped on a cold glass slide and air-dried. Slides were stained with 5% Giemsa solution and encoded. Two hundred metaphases per substance (100 metaphases from each slide) were observed under the microscope (at magnification of 1000 X). The percentage of aberrant cells per subject was recorded according to the type of aberration.

### In vivo alkaline comet assay

#### Test animals

All animal studies were approved by the Institutional Animal Care and Use Committee of Seoul National University (approval number SNU-201215-3-1). Five-week-old Hsd: ICR (CD-1) male mice were supplied by DooYeol Biotech, Korea, and housed up to 5 per cage at a room temperature of 23 ± 1 °C with a relative humidity of 50 ± 10% and a 12 h light/dark cycle. Diet and drinking water were available ad libitum.

#### Experimental design and treatment

The experimental design and exposure doses were set according to the OECD 489 test guideline [42] and an international validation study of the in vivo rodent alkaline comet assay coordinated by the Japanese Centre for the Validation of Alternative Methods [43]. After 5 days of acclimation, 4 groups of male mice (*n* = 5/dose group) were dosed via oral gavage with either 0 (vehicle control), 500, 1000, or 2000 mg/kg b.w./day of the test substances (*gardenia* yellow, *Curcuma* extract, *Monascus* red) in deionized water (vehicle) at a dose volume of 10 ml/kg b.w. at 0 h (day 1), 24 h (day 2), and 45 h (day 3). The preliminary study showed that oral administration of these substances at a dose of 2000 mg/kg b.w./day did not induce animal death. Animals in the positive control group (*n* = 3) were dosed via oral gavage with ethyl methanesulfonate (200 mg/kg b.w./day) at 24 h (day 2) and 45 h (day 3). Animals were euthanized by cardiac puncture under isoflurane anesthesia 2–6 h after the last test dose.

#### Isolation of single-cell suspensions for the comet assay

Single cell suspensions from the liver and stomach were isolated based on the JaCVAM [43] and OECD TG 489 [42] methods. The livers were rinsed with cold mincing solution (MS; Hanks’ Balanced Salt Solution with 20 mM EDTA, 10% DMSO, pH 7.0–7.5) until no blood was visible and then finely cut (minced) with a pair of fine scissors and placed in a 1.7 ml tube containing up to 1 ml of chilled MS to release the cells. Stomachs were washed free of food using cold MS, and the forestomach was removed and discarded. The glandular stomach was placed into fresh buffer and incubated on ice for 15 minutes. After incubation, the surface layer was gently scraped two times with a cell scraper and discarded. The stomach epithelia were carefully scraped 4–5 times to release the cells. The cells released from each tissue were strained through a 40 μm cell strainer, and the resulting suspension was used to prepare the comet slides.

#### Alkaline comet assay

The liver and stomach cell suspension was mixed with 0.5% low melting point agarose gel (ratio 1:9 volume fraction) and applied to commercially available pre-treated 2-well microscope slides (Trevigen, Inc). The slides were kept at 4 °C for at least 5 min to allow the gel to solidify, and then they were immersed in chilled lysis solution (2.5 M NaCl, 100 mM EDTA, 10 mM tris hydroxymethyl aminomethane, 1% Triton X-100, 10% DMSO, pH 10) overnight in a refrigerator to facilitate removal of the cell membrane and histone. After cell lysis, the slides were washed with distilled water and transferred to a freshly prepared alkaline solution (300 mM NaOH, 1 mM EDTA, pH > 13). The slides were incubated in this solution for 20 min at room temperature to allow DNA to unwind. Electrophoresis was conducted in the same buffer for 30 min at 0.7 V/cm and 2–10 °C. After the end of electrophoresis, the slides were immersed in a neutralization buffer (0.4 M tris hydroxymethyl aminomethane in purified water, pH 7.5) for at least 5 min, rinsed with distilled water twice, and fixed for 5 min in absolute ethanol. Then the slides were air-dried for 15 min at 37 °C and stained with SYBR-gold™. The comets were measured using a fluorescence microscope at a magnification of 200X. One hundred randomly selected images per animal were scored for DNA damage using Comet Assay IV image analysis software (Perceptive Instruments Ltd., UK).

### Statistical analysis

Statistical analysis of the data was performed using IBM SPSS statistics v25. For the bacterial reverse mutation assay and cell viability test, the data were analyzed using one-way analysis of variance, and the significance of inter-group differences was analyzed using Tukey’s test. For the mammalian chromosomal aberration assay, chi-square and Fisher’s exact tests were used to identify significant differences in aberrant metaphase cell frequency between the treated and negative control groups. A difference was considered significant when the *p*-value was < 0.05. For the alkaline comet assay, Dunnett’s test (two-sided, *p* < 0.05) and the linear trend test (two-sided, *p* < 0.05) were used to assess differences between the treated and vehicle control groups. The positive control group was compared to the vehicle control group using Student’s t-test (one-sided, *p* < 0.025).

## Results

### Bacterial reverse mutation assay

The results of the bacterial reverse mutation assay are shown in Table 1. The mutagenicity result for each group was considered positive if the number of revertant colonies reached a minimum of two-fold the negative control with a dose relationship in at least one strain [37, 44, 45]. *Gardenia* yellow was found to have a mutagenic effect on *S. typhimurium* TA98 in the presence of the S9 mixture. In *S. typhimurium* TA1537 and TA100, revertant colonies increased significantly but not by two-fold. *Curcuma* extract exhibited an abundant of microcolonies in *S. typhimurium* TA1537 with the S9 mixture and in case of *S. typhimurium* TA100 without the S9 mixture. In *S. typhimurium* TA100 in the absence of the S9 mixture, microcolonies observed at a dose of 500 μg/plate decreased as concentration increases. *Monascus* red exposure led to microcolonies at a dose of 5000 μg/plate in *S. typhimurium* TA1537 and TA100 in the absence of the S9 mixture. *Curcuma* extract and *Monascus* red did not show mutagenic effects at concentrations where they did not produce microcolonies. *Gardenia* red and blue, cochineal extract, lac color, and beet red tested negative for mutagenicity in all test strains. The number of revertant colonies in the positive controls was within the range of the historical positive control data collected by Kato et al. from 2013 to 2016 [46].

**Table 1:** Bacterial reverse mutation assay results for natural color additives

Test Substance	Conc. (μg/plate)	Absence of metabolic system															Presence of metabolic system														
		Frameshift mutation						Base-pair substitution									Frameshift-sensitive						Base-pair substitution								
		TA98			TA1537			TA100			TA1535			*E.Coli*			TA98			TA1537			TA100			TA1535			*E.Coli*		
*Gardenia* Red	0	24	±	3	14	±	2	125	±	13	16	±	5	51	±	1	36	±	1	31	±	1	174	±	2	13	±	3	48	±	4
	50	20	±	4	15	±	6	127	±	15	13	±	2	41	±	4	38	±	2	28	±	2	188	±	9	18	±	4	52	±	4
	150	23	±	3	12	±	2	132	±	15	14	±	3	48	±	10	39	±	1	23	±	8	175	±	5	15	±	2	61	±	5^*^
	500	25	±	9	12	±	3	145	±	8	16	±	2	44	±	6	35	±	2	32	±	2	167	±	12	15	±	4	48	±	5
	1500	25	±	3	10	±	4	143	±	12	13	±	2	44	±	7	33	±	7	28	±	6	181	±	12	16	±	1	52	±	3
	5000	19	±	4	12	±	3	145	±	1	15	±	5	46	±	6	35	±	7	29	±	2	162	±	18	12	±	5	48	±	6
*Gardenia* Yellow	0	22	±	1	12	±	2	139	±	22	13	±	5	46	±	8	36	±	4	28	±	1	134	±	1	13	±	6	50	±	2
	50	23	±	5	11	±	1	129	±	33	17	±	5	54	±	4	37	±	5	33	±	9	149	±	9	16	±	3	49	±	6
	150	21	±	2	12	±	3	139	±	5	12	±	3	53	±	1	33	±	4	32	±	5	150	±	4	14	±	1	54	±	6
	500	31	±	2^**^	10	±	3	130	±	6	15	±	4	50	±	4	42	±	5	33	±	8	143	±	9	14	±	3	51	±	6
	1500	24	±	2	12	±	3	130	±	4	12	±	3	53	±	5	43	±	3	37	±	9	152	±	8	13	±	3	57	±	11
	5000	37	±	1^***^	12	±	1	136	±	15	15	±	6	46	±	14	85	±	4^***^	50	±	5^*^	163	±	9^**^	12	±	3	50	±	4
*Gardenia* Blue	0	20	±	3	13	±	1	149	±	11	16	±	4	50	±	9	35	±	10	28	±	4	167	±	25	23	±	1	42	±	5
	50	20	±	5	13	±	2	167	±	9	26	±	3^**^	52	±	5	33	±	12	22	±	5	169	±	19	22	±	4	44	±	5
	150	22	±	2	9	±	2	164	±	13	24	±	3	45	±	5	28	±	10	25	±	4	155	±	14	22	±	6	46	±	10
	500	24	±	7	10	±	4	160	±	13	23	±	3	54	±	5	30	±	8	21	±	2	165	±	4	17	±	2	45	±	8
	1500	21	±	5	12	±	3	154	±	7	28	±	1^**^	51	±	7	32	±	3	20	±	2	145	±	11	23	±	2	48	±	11
	5000	34	±	8	12	±	3	143	±	11	27	±	3^**^	45	±	3	33	±	6	23	±	4	114	±	5^*^	24	±	5	45	±	6
Cochineal Extract	0	20	±	3	15	±	2	119	±	4	10	±	1	53	±	8	33	±	11	39	±	22	235	±	41	13	±	1	46	±	5
	50	24	±	2	15	±	2	123	±	5	13	±	2	55	±	6	28	±	8	43	±	7	224	±	27	12	±	4	55	±	2
	150	19	±	4	21	±	3	118	±	1	14	±	3	43	±	9	32	±	2	39	±	7	216	±	14	15	±	1	57	±	5
	500	20	±	1	18	±	6	122	±	10	16	±	5	51	±	2	30	±	9	43	±	11	219	±	26	12	±	4	49	±	3
	1500	23	±	6	19	±	8	141	±	1^**^	13	±	1	45	±	13	33	±	6	46	±	3	217	±	25	17	±	5	55	±	5
	5000	18	±	5	19	±	7	151	±	8^***^	10	±	4	52	±	4	32	±	1	40	±	4	230	±	8	10	±	2	54	±	7
Lac Color	0	26	±	5	22	±	6	123	±	12	12	±	4	44	±	3	33	±	2	29	±	4	190	±	16	14	±	4	52	±	4
	50	22	±	2	27	±	3	135	±	4	15	±	2	55	±	2	46	±	5	30	±	2	177	±	19	17	±	8	53	±	4
	150	23	±	4	27	±	2	125	±	6	13	±	4	54	±	9	31	±	4	37	±	9	181	±	9	16	±	7	52	±	9
	500	26	±	8	26	±	6	127	±	9	13	±	2	54	±	7	35	±	3	28	±	1	173	±	7	16	±	4	54	±	2
	1500	20	±	3	26	±	3	143	±	6	13	±	5	58	±	4	33	±	9	27	±	4	184	±	10	15	±	4	50	±	6
	5000	23	±	5	23	±	3	132	±	9	14	±	2	49	±	6	34	±	6	34	±	5	165	±	20	16	±	7	52	±	10
Beet Red	0	21	±	4	16	±	3	142	±	23	11	±	2	59	±	8	35	±	3	45	±	11	173	±	1	13	±	3	47	±	2
	50	19	±	1	16	±	1	148	±	11	14	±	4	50	±	5	40	±	10	34	±	3	170	±	10	14	±	2	50	±	5
	150	20	±	2	16	±	5	161	±	15	14	±	3	53	±	6	37	±	10	38	±	2	174	±	14	11	±	6	52	±	10
	500	27	±	2	19	±	7	148	±	12	16	±	3	53	±	5	38	±	6	38	±	6	169	±	12	10	±	4	50	±	5
	1500	19	±	7	20	±	2	153	±	21	14	±	7	56	±	4	31	±	7	39	±	6	179	±	4	15	±	3	52	±	4
	5000	20	±	4	17	±	3	171	±	7	14	±	5	56	±	7	35	±	4	38	±	2	183	±	18	15	±	4	50	±	6
*Curcuma* extract	0	22	±	5	15	±	2	121	±	8	15	±	6	57	±	5	26	±	1	48	±	12	140	±	7	20	±	3	55	±	4
	50	24	±	7	15	±	1	132	±	8	17	±	5	57	±	6	28	±	3	44	±	12	156	±	13	14	±	2	52	±	2
	150	29	±	1	13	±	6	116	±	6	15	±	6	60	±	2	31	±	4	61	±	38	165	±	9	14	±	4	49	±	5
	500	26	±	4	14	±	3	microcolonies^a^			16	±	4	53	±	7	32	±	8	36	±	7	147	±	13	15	±	4	54	±	6
	1500	23	±	10	microcolonies^a^			869	±	68^***^	8	±	4	44	±	4	37	±	6	32	±	10	133	±	7	14	±	3	50	±	10
	5000	24	±	6	microcolonies^a^			142	±	27	10	±	3	54	±	7	29	±	1	28	±	9	microcolonies^a^			8	±	3^**^	46	±	3
*Monascus* Red	0	21	±	6	14	±	3	148	±	11	14	±	1	51	±	13	29	±	5	39	±	1	199	±	13	15	±	1	45	±	1
	50	22	±	2	16	±	4	166	±	12	13	±	2	51	±	7	33	±	3	31	±	4	203	±	16	19	±	6	47	±	6
	150	23	±	4	14	±	4	172	±	3	16	±	2	60	±	8	35	±	10	38	±	6	191	±	28	24	±	3	50	±	9
	500	16	±	3	16	±	2	137	±	20	14	±	3	50	±	7	34	±	3	34	±	2	167	±	16	14	±	2	49	±	14
	1500	19	±	1	8	±	4	144	±	15	14	±	2	50	±	8	33	±	7	3	±	9	157	±	7	16	±	6	49	±	3
	5000	23	±	5	microcolonies^a^			microcolonies^a^			15	±	2	55	±	10	38	±	2	28	±	6	159	±	14	18	±	2	54	±	6
Positive control		156	±	68^b^	119	±	29^c^	456	±	86^b^	358	±	29^d^	151	±	16^e^	790	±	395^f^	205	±	106^g^	1277	±	370^h^	218	±	75^g^	290	±	121^i^

Data are presented as the mean ± SD of three replicates

^*^p < 0.05, ^**^p < 0.01 or ^***^p < 0.001 compared to the negative control (0 μg/plate)

^a^Each plate was divided into 16 sections, and each section contained 300 or more microcolonies

^b^2-(2-furyl)-3-(5-nitro-2-furyl) acylamide (AF-2) 0.1 μg/plate, ^c^9-Aminoacridine (9-AA) 40 μg/plate, ^d^Sodium azide (NaN3) 0.5 μg/plate,

^e^AF-2 0.01 μg/plate, ^f^2-Aminoanthracene (2-AA) 0.5 μg/plate, ^g^2-AA 2 μg/plate, ^h^: 2-AA 1 μg/plate, ^i^2-AA 10 μg/plate

### Cytotoxicity test

**Fig. 1:**
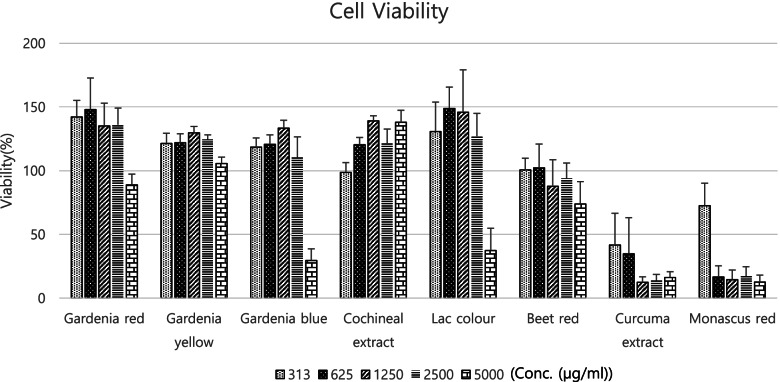
Cell viability of eight natural color additives on MTT assay. Eight color additives were treated to CHO-K1 cells. Data are expressed as the mean ± SD

Among the eight natural color additives, *gardenia* red and yellow, cochineal extract, and beet red showed no cytotoxicity at the maximum test concentration of 5000 μg/ml on MTT assay. *Gardenia* blue and lac color showed 50% or higher inhibition of cell survival at a concentration of 5000 μg/ml, and *Curcuma* extract and *Monascus* red showed toxicities at concentrations higher than 313 μg/ml (Fig. 1). The IC_50_ of the inhibition of cell viability was 3761 μg/ml, 2484 μg/ml, 420 μg/ml, and 786 μg/ml for *gardenia* blue, lac color, *Monascus* red, and *Curcuma* extract, respectively.

### In vitro chromosomal aberration test

The maximum concentration of test substances was determined based on the cytotoxicity test. Duplicate samples of 100 cells per plate were observed in metaphase and classified for numerical abnormalities (polyploids and endoreduplication) and structural abnormalities (chromatid breaks, chromatid exchanges, chromosomal breaks, and chromosomal exchanges). The results were deemed positive only when the percentage of chromosomal aberrations was ≥10% by the criteria of Galloway et al. [47]. There was no statistically significant difference in the frequency of cells with chromosomal aberrations at any dose level for any test substance except *Curcuma* extract and *Monascus* red. In the case of *Curcuma* extract and *Monascus* red, a statistically significant increase in total cells with chromosomal aberrations was observed at concentration of 313 μg/ml in a short-duration treatment without metabolic activation (Tables 2, 3 and 4). Although *Curcuma* extract and *Monascus* red exhibited a statistically significant difference (*p* < 0.05) in the frequency of cells with chromosomal aberrations, this result was interpreted as weak positive because the percentage of chromosomal aberrations did not exceed 10%.

**Table 2:** Results of the in vitro chromosome aberration test with short-term treatment and without metabolic activation

Test substance	Conc. (μg/ml)	Obs. Cell No.	S9 mix	Trt-Rec Time (hrs)	Number of chromosomal aberrations									
					Numerical aberrations				Structural aberrations					
					Pol	Endo	Total	(%)	ctb	cte	csb	cse	Total	(%)
Negative Control		200	–	6–18	0	0	0	(0)	1	0	0	0	1	(0.5)
*Gardenia* Red	313				0	0	0	(0)	0	0	0	0	0	(0)
	625				0	0	0	(0)	0	0	0	0	0	(0)
	1250				0	0	0	(0)	1	0	0	0	1	(0.5)
	2500				0	0	0	(0)	1	0	0	0	1	(0.5)
	5000				0	0	0	(0)	2	0	0	0	2	(1)
*Gardenia* Yellow	313				0	0	0	(0)	2	0	0	0	2	(1)
	625				0	0	0	(0)	1	0	0	0	1	(0.5)
	1250				0	0	0	(0)	0	0	0	0	0	(0)
	2500				0	0	0	(0)	0	0	0	0	0	(0)
	5000				0	0	0	(0)	0	0	0	0	0	(0)
*Gardenia* Blue	313				0	0	0	(0)	0	0	0	0	0	(0)
	625				0	0	0	(0)	2	0	0	0	2	(1)
	1250				0	0	0	(0)	0	0	0	0	0	(0)
	2500				0	0	0	(0)	0	0	0	0	0	(0)
Cochineal Extract	313				0	0	0	(0)	0	0	0	0	0	(0)
	625				0	0	0	(0)	1	0	0	0	1	(0.5)
	1250				0	0	0	(0)	1	0	0	0	1	(0.5)
	2500				0	0	0	(0)	0	0	0	0	0	(0)
	5000				0	0	0	(0)	1	0	0	0	1	(0.5)
Lac Color	313				0	0	0	(0)	0	0	0	0	0	(0)
	625				0	0	0	(0)	1	0	0	0	1	(0.5)
	1250				0	0	0	(0)	0	0	0	0	0	(0)
	2500				0	0	0	(0)	0	0	0	0	0	(0)
Beet Red	313				0	0	0	(0)	3	0	0	0	3	(1.5)
	625				0	1	1	(0.5)	2	0	0	0	2	(1.0)
	1250				0	0	0	(0)	1	0	0	0	1	(0.5)
	2500				0	0	0	(0)	2	0	0	0	2	(1)
	5000				0	0	0	(0)	1	0	0	0	1	(0.5)
*Curcuma* extract	78				0	0	0	(0)	4	0	0	0	4	(2)
	156				0	0	0	(0)	3	0	0	0	3	(1.5)
	313				0	0	0	(0)	8	0	0	0	8^*^	(4)
*Monascus* Red	78				0	0	0	(0)	1	0	0	0	1	(0.5)
	156				0	0	0	(0)	1	0	1	0	2	(1)
	313				1	0	1	(0.5)	6	0	2	0	8^*^	(4.0)
Positive control^a^					0	0	0	(0)	21	9	6	0	36^*^	(18)

Negative control: sterile distilled water

Positive control: 4-nitroquinoline-N-oxide 1.0 μg/ml

*Trt-Rec Time* treatment-recovery times

*Pol* polyploids, *Endo* endoreduplication, *ctb* chromatid breakage, *cte* chromatid exchange, *csb* chromosome breakage, *cse* chromosome exchange

^*^: Significantly different from the negative control by Fisher’s exact test at *p* < 0.05

**Table 3:** Results of the in vitro chromosome aberration test with short-term treatment and with metabolic activation

Test substance	Conc. (μg/ml)	Obs. Cell No.	S9 mix	Trt-Rec Time (hrs)	Number of chromosomal aberrations									
					Numerical aberrations				Structural aberrations					
					Pol	Endo	Total	(%)	ctb	cte	csb	cse	Total	(%)
Negative Control		200	+	6–18	0	0	0	(0)	2	0	0	0	2	(1)
*Gardenia* Red	313				0	0	0	(0)	2	0	0	0	2	(1)
	625				0	0	0	(0)	2	0	0	0	2	(1)
	1250				0	0	0	(0)	3	0	0	0	3	(1.5)
	2500				0	0	0	(0)	0	0	0	0	0	(0)
	5000				0	0	0	(0)	4	0	0	0	4	(2)
*Gardenia* Yellow	313				0	0	0	(0)	0	0	0	0	0	(0)
	625				0	0	0	(0)	0	0	0	0	0	(0)
	1250				0	0	0	(0)	0	0	0	0	0	(0)
	2500				0	0	0	(0)	0	0	0	0	0	(0)
	5000				0	0	0	(0)	1	0	0	0	1	(0.5)
*Gardenia* Blue	313				0	0	0	(0)	0	0	0	0	0	(0)
	625				0	0	0	(0)	0	0	0	0	0	(0)
	1250				0	0	0	(0)	0	0	0	0	0	(0)
	2500				0	0	0	(0)	0	0	0	0	0	(0)
Cochineal Extract	313				0	0	0	(0)	0	0	0	0	0	(0)
	625				0	0	0	(0)	1	0	0	0	1	(0.5)
	1250				0	0	0	(0)	0	0	0	0	0	(0)
	2500				0	0	0	(0)	1	0	0	0	1	(0.5)
	5000				0	0	0	(0)	1	0	0	0	1	(0.5)
Lac Color	313				0	0	0	(0)	0	0	0	0	0	(0)
	625				0	0	0	(0)	3	0	0	0	3	(1.5)
	1250				0	0	0	(0)	1	0	0	0	1	(0.5)
	2500				0	0	0	(0)	1	0	0	0	1	(0.5)
Beet Red	313				0	0	0	(0)	2	0	0	0	2	(1)
	625				0	0	0	(0)	2	0	2	0	4	(2)
	1250				0	0	0	(0)	2	0	0	0	2	(1)
	2500				0	0	0	(0)	4	0	1	0	5	(2.5)
	5000				0	0	0	(0)	2	1	0	0	3	(1.5)
*Curcuma* extract	78				0	0	0	(0)	1	0	0	0	1	(0.5)
	156				0	0	0	(0)	4	0	0	0	4	(2)
	313				1	1	2	(1)	8	1	0	0	9	(4.5)
*Monascus* Red	78				0	0	0	(0)	4	0	2	0	6	(3)
	156				0	0	0	(0)	5	0	1	1	7	(3.5)
	313				0	2	2	(1)	4	0	0	0	4	(2)
Positive control					0	0	0	(0)	45	16	24	1	86^*^	(43)

Negative control: sterile distilled water

Positive control: cyclophosphamide monohydrate 5.0 μg/ml

*Trt-Rec Time* treatment-recovery times

*Pol* polyploids, *Endo* endoreduplication, *ctb* chromatid breakage, *cte* chromatid exchange, *csb* chromosome breakage, *cse* chromosome exchange

^*^: Significantly different from the negative control by Fisher’s exact test at *p* < 0.05

**Table 4:** Results of the in vitro chromosome aberration test with continuous treatment and without metabolic activation

Test substance	Conc. (μg/ml)	Obs. Cell No.	S9 mix	Trt-Rec Time (hrs)	Number of chromosomal aberrations									
					Numerical aberrations				Structural aberrations					
					Pol	Endo	Total	(%)	ctb	cte	csb	cse	Total	(%)
Negative Control		200	–	24–0	0	0	0	(0)	2	0	0	0	2	(1)
*Gardenia* Red	313				0	0	0	(0)	2	1	0	0	3	(1.5)
	625				0	0	0	(0)	2	0	0	0	2	(1)
	1250				0	0	0	(0)	0	0	0	0	0	(0)
	2500				0	0	0	(0)	0	0	0	0	0	(0)
	5000				0	0	0	(0)	1	0	0	0	1	(0.5)
*Gardenia* Yellow	313				0	0	0	(0)	0	0	0	0	0	(0)
	625				0	0	0	(0)	0	0	0	0	0	(0)
	1250				0	0	0	(0)	0	0	0	0	0	(0)
	2500				0	0	0	(0)	1	0	0	0	1	(0.5)
	5000				0	0	0	(0)	1	0	0	0	1	(0.5)
*Gardenia* Blue	313				0	0	0	(0)	0	0	0	0	0	(0)
	625				0	0	0	(0)	0	0	0	0	0	(0)
	1250				0	0	0	(0)	1	0	0	0	1	(0.5)
	2500				0	0	0	(0)	0	0	0	0	0	(0)
Cochineal Extract	313				0	0	0	(0)	0	0	0	0	0	(0)
	625				0	0	0	(0)	0	0	0	0	0	(0)
	1250				0	0	0	(0)	0	0	0	0	0	(0)
	2500				0	0	0	(0)	0	0	0	0	0	(0)
	5000				0	0	0	(0)	1	0	0	0	1	(0.5)
Lac Color	313				0	0	0	(0)	1	0	0	0	1	(0.5)
	625				0	0	0	(0)	0	0	0	0	0	(0)
	1250				0	0	0	(0)	1	0	0	0	1	(0.5)
	2500				0	0	0	(0)	2	0	0	0	2	(1)
Beet Red	313				0	0	0	(0)	3	0	0	0	3	(1.5)
	625				0	0	0	(0)	0	0	0	0	0	(0)
	1250				0	0	0	(0)	1	0	0	0	1	(0.5)
	2500				0	0	0	(0)	1	1	0	0	2	(1)
	5000				0	0	0	(0)	1	0	0	0	1	(0.5)
*Curcuma* extract	78				0	0	0	(0)	1	0	0	0	1	(0.5)
	156				0	0	0	(0)	4	0	0	0	4	(2)
	313				0	0	0	(0)	7	0	1	0	8	(4)
*Monascus* Red	78				0	0	0	(0)	3	0	1	0	4	(2)
	156				0	0	0	(0)	2	0	0	0	2	(1)
	313				0	1	1	(0.5)	7	0	2	0	9	(4.5)
Positive control					1	1	2	(1)	29	0	1	2	32^*^	(16)

Negative control: sterile distilled water

Positive control: 4-nitroquinoline-N-oxide 0.5 μg/ml

*Trt-Rec Time* treatment-recovery times

*Pol* polyploids, *Endo* endoreduplication, *ctb* chromatid breakage, *cte* chromatid exchange, *csb* chromosome breakage, *cse* chromosome exchange

^*^: Significantly different from the negative control by Fisher’s exact test at *p* < 0.05

### In vivo alkaline comet assay

**Fig. 2:**
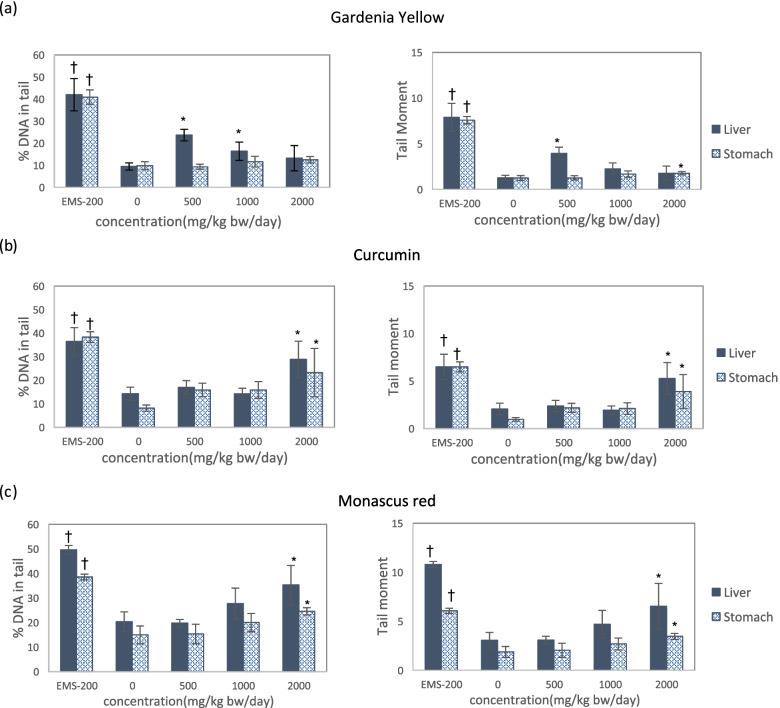
The level of DNA damage in the livers and stomachs of male ICR mice, as measured by the alkaline comet assay. The DNA damage induced by *gardenia* yellow (**a**), *Curcuma* extract (**b**), and *Monascus* red (**c**). The results are expressed as the mean ± SD of three independent experiments. * *p* < 0.05 (Dunnett’s post-hoc test, two-sided) is considered significantly different from the negative control group values. ^†^*p* < 0.025 (Student’s t-test, one-sided) is considered significantly different from the negative control

*Gardenia* yellow showed statistically significant increases in DNA damage in liver cells at concentrations of 500 mg/kg b.w./day and 1000 mg/kg b.w./day, but the increases were not dose dependent (Fig. 2). When both criteria are satisfied, the substance is judged to be positive; therefore, *gardenia* yellow was not considered positive in the alkaline comet assay. However, oral administration of either *Curcuma* extract or *Monascus* red produced statistically significant increases in DNA damage in both liver and stomach cells at the maximum test concentration of 2000 mg/kg b.w./day (Fig. 2). Figure 2 shows the linear dependency of the effects of *Curcuma* extract and *Monascus* red on concentration (*p* < 0.05). These results indicate positivity in the in vivo comet assay. The positive control also showed DNA damage.

## Discussion

Despite the frequent use of natural color additives, few scientific studies have been undertaken to determine their safety under the pretext of ‘a history of safe use’. However, history alone cannot guarantee the absence of genotoxicity. Therefore, we conducted genotoxicity testing as a minimum step to confirm the safety of natural color additives. We conducted genotoxicity tests for *gardenia* red, blue, and yellow; lac color; cochineal extract; beet red; *Curcuma* extract, and *Monascus* red, which are all permitted as food colorants in Korea.

Genotoxicity testing requires a battery of tests because no single test can cover the various genotoxic endpoints. Therefore, to evaluate the potential genotoxicity of natural color additives in this study, we performed a bacterial reverse mutation test (Ames test) using *S. typhimurium* and *E. coli* to detect gene mutation, a chromosomal aberration test in CHO-K1 cells to detect numerical and structural chromosomal alterations, and an in vivo alkaline comet assay.

We found no positive responses to the *gardenia* red and blue, cochineal extract, lac color, or beet red in the Ames test or in vitro chromosomal aberration test compared with the concurrent vehicle control groups both with and without the application of S9. Although the MSDF recommends a standard battery of three tests (bacterial reverse mutation test, one in vitro test, and one in vivo test), the EFSA recommends a step-wise approach composed of in vitro assays for initial genotoxicity evaluations, except in some cases. In the step-wise approach, if the basic in vitro battery test yields a negative result, the test substances are deemed to non-genotoxic [22]. Therefore, we concluded that those five food colors are without genotoxic potential without conducting further experiments.

When substances show genotoxic potential in the in vitro tests, follow-up in vivo tests can provide additional information about human health effects [22]. In this study, the in vitro genotoxicity studies produced equivocal results about the genotoxic potential of *gardenia* yellow, *Curcuma* extract, and *Monascus* red. As a follow-up study, we selected the in vivo alkaline comet assay, which is considered to be sensitive for substances that produce gene mutations or structural chromosomal aberrations and can be applied to various target organs [22, 48].

The *Gardenia* yellow tested in the present study was mutagenic in the Ames test, but it was not positive in the in vitro chromosomal aberration test nor in vivo alkaline comet assay. Therefore, *Gardenia* yellow was not considered a genotoxic substance.

**Fig. 3:**
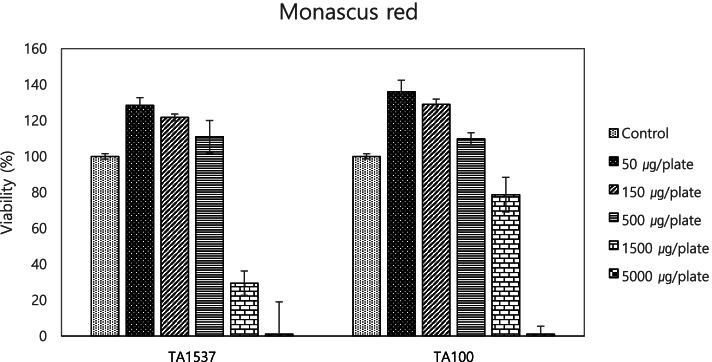
Viability of bacteria treated by *Monascus* red. Data are expressed as the mean ± SD

In the case of *Monascus* red, microcolonies were observed at a concentration of 5000 μg/plate in the Ames test (Table 1), and an increase in revertant colony was not observed at concentrations that did not produce microcolonies. Also, when bacterial growth was evaluated in nutrient broth, inhibition was observed at concentrations of 1500 μg/plate and above for *Monascus* red (Fig. 3). Because microcolonies can be considered a consequence of cytotoxicity [49], we concluded that *Monascus* red was not mutagenic. In the in vitro mammalian cell mutagenicity test, *Monascus* red showed a statistically significant increase in cells with chromosomal abnormalities only at maximum concentration, but the percentage of chromosomal aberrations did not exceed 10%. The colorant induced a statistically significant increase in % DNA in the tail and tail moment at maximum concentration and showed dose dependency in the in vivo comet assay. The weak positive result on a mammalian cell in vitro mutagenicity testing and the dose dependency of the in vivo comet test results suggest probable genotoxicity. In Korea, no ADI has been set for *Monascus* red due to a lack of toxicity data, including genotoxicity, in addition to its natural origin [27]. The basic toxicological data required for a scientifically sound safety evaluation were not available [50]. Therefore, the results of our toxicity testing of *Monascus* should trigger further investigation and reevaluation of the safety of this natural colorant.

For *Curcuma* extract, microcolony observation in the Ames test could be considered a consequence of cytotoxicity; therefore, we concluded that *Curcuma* extract was not a bacterial mutagen. However, we observed statistically significant increases in both the in vitro and in vivo assays using mammalian systems, and we found a statistically significant linear correlation between % DNA in the tail intensity/tail moment and the concentration of *Curcuma* extract in the in vivo comet assay. Therefore, *Curcuma* extract was suspected of having genotoxic potential in this study.

The JECFA concluded that *Curcuma* extract was not carcinogenic based on the National Toxicology Program technical report. In chronic toxicity/carcinogenicity studies using B6C3F1 mice for 103 weeks, no significant differences in hematological or clinical chemistry were found between the control and treated groups. Although there was an increase in the incidence of hepatocellular adenoma and small intestine carcinomas, JECFA determined them to be equivocal evidence. In addition, the increases in the occurrence of clitoral gland adenomas observed in F344/N female rats were not dose-dependent. As a result, JECFA set an ADI of 0–3 mg/kg b.w./day based on the No Observed Adverse Effect Level (NOAEL) of the reproductive toxicity [33, 51]. Although the EFSA expressed no concern regarding genetic toxicity for *Curcuma* extract, their panel did not disregard the positive results for *Curcuma* extract in several genotoxicity tests. *Curcuma* extract tested positive in the *rec* assay and in vitro chromosomal aberration assay and induced DNA damage in human lymphocytes, Chinese hamster ovary cells, and HepG2 cells [34]. In an in vivo study, it caused a statistically significant, dose-dependent increase in micronuclei frequency and total chromosomal aberration frequency [34]. Though the results from these study are also equivocal, since the functionality of the *Curcuma* extract is widely referred, more studies of *Curcuma* extract’s toxicity are warranted [52, 53].

Regardless of its genotoxicity, we found *Monascus* red and *Curcuma* extract to be cytotoxic to all the types of cells used in this study. *Monascus* red and *Curcuma* extract showed cytotoxicity on the Ames test, and cell viability was less than 50% at the concentration of higher than 313 μg/ml in our cell viability tests using CHO-K1 cells. Also, we found several non-analyzable morphological defects caused by cytotoxicity in the comet images of *Curcuma* extract-treated liver and stomach cells. These results suggest that the cytotoxicity of *Monascus* red and *Curcuma* extract should be evaluated further to ensure its safety. In addition, to more accurately interpret the cytotoxicity of *Monascus* red and *Curcuma* extract, further studies on its mechanism are needed.

The present study was designed to assure the safety of the colorants in the current market, and available products were used as test substances. Since natural colorants are mostly extracts and sold as mixtures, an undefined chemical composition is the limitation of the study. Follow-up studies should be carried out to identify the causative compounds in colorants that were suspected of being genotoxic in the present study.

## Conclusion

Eight natural food colors that are commercially available in the Korean market were selected for genotoxicity tests. Among them, *gardenia* red, blue, and yellow; cochineal extract; lac color; and beet red did not show any genotoxicity. *Curcuma* extract and *Monascus* red showed definite cytotoxicity and probable genotoxicity. Because few toxicological studies have been conducted on natural color additives, it is necessary to develop a toxicological database that will include results from additional tests for long- and short-term toxicity, carcinogenicity, and reproductive toxicity. This will help to ensure the safety of frequently-consumed natural compounds with a history of use, and to set regulatory values for these additives.

## Data Availability

All data generated or analyzed during this study are included in this published article.
